# Induction of Germ Cell-like Cells from Porcine Induced Pluripotent Stem Cells

**DOI:** 10.1038/srep27256

**Published:** 2016-06-06

**Authors:** Hanning Wang, Jinzhu Xiang, Wei Zhang, Junhong Li, Qingqing Wei, Liang Zhong, Hongsheng Ouyang, Jianyong Han

**Affiliations:** 1 State Key Laboratories for Agrobiotechnology, College of Biological Sciences, China Agricultural University, Beijing, 100193, China; 2 Jilin Provincial Key Laboratory of Animal Embryo Engineering, College of Animal Sciences, Jilin University, 5333 Xi’an Road, Changchun, Jilin, 130062, China

## Abstract

The ability to generate germ cells from pluripotent stem cells (PSCs) is valuable for human regenerative medicine and animal breeding. Germ cell-like cells (GCLCs) have been differentiated from mouse and human PSCs, but not from porcine PSCs, which are considered an ideal model for stem cell applications. Here, we developed a defined culture system for the induction of primordial germ cell-like cells (PGCLCs) from porcine induced PSCs (piPSCs). The identity of the PGCLCs was characterized by observing cell morphology, detecting germ cell marker gene expression and evaluating epigenetic properties. PGCLCs could further differentiate into spermatogonial stem cell-like cells (SSCLCs) *in vitro*. Importantly, meiosis occurred during SSCLC induction. Xenotransplantation of GCLCs into seminiferous tubules of infertile immunodeficient mice resulted in immunohistochemically identifiable germ cells *in vivo*. Overall, our study provides a feasible strategy for directing piPSCs to the germ cell fate and lays a foundation for exploring germ cell development mechanisms.

The germ cell lineage, which is the source of totipotency, is capable of differentiating into all types of cells. Errors in germ cell derivation and differentiation lead to negative outcomes that include infertility, tumors, or even congenital defects in offspring. Therefore, the derivation of germ cells from PSCs has potential applications in the study of germ cell development mechanisms and infertility treatments.

Great progress has been made in the induction of PGCLCs from mouse and human PSCs. Direct induction of mouse and human PSCs into PGCLCs, spermatogonia, and even mature gametes, has been reported. In mice, a defined culture system including growth factors has been established for the differentiation of PGCLCs from PSCs^1^,^2^,^3^. Without the cytokines, the combination of transcription factors (TFs) PRDM1, PRDM14 and TFAP2C induces the primordial germ cell (PGC) fate because the TFs are necessary for PGC specification during mouse *in vivo* development^4^,^5^,^6^. Previous studies have also demonstrated that retinoic acid (RA) acts to promote the differentiation of embryonic stem cells (ESCs) into germ cells, and even functional gametes, which can initiate and support *in vivo* development after intracytoplasmic injection (ICSI)^7^,^8^. The entrance into meiosis is stimulated by RA and mediated by the *Stra8* gene^9^. Moreover, overexpression of Deleted in Azoospermia-Like (*Dazl*) directs ESCs into gametes *in vitro*^10^. Recently, mouse ESC-derived PGCLCs have completed meiosis *in vitro*^11^.

Human PGCLCs have also been generated from PSCs upon stimulation with BMP4, SCF and EGF^12^,^13^,^14^. Divergent early postimplantation development and PSC regulation may result in the mechanistic differences between human and mouse PGC specification. SOX17 is a key specifier of human PGC fate and a marker of human PGCLCs, which is not the case in mouse PGC specification^12^. In addition, human PSCs can be induced into SSCLCs, enter meiosis, and differentiate into haploid spermatogenic cells *in vitro* by overexpressing *Dazl* and other related genes or adding growth factors such as RA to the culture systems^15^,^16^,^17^,^18^. Transplantation of human iPSCs directly into mouse seminiferous tubules, which provide a germ cell niche, can direct the germ cell differentiation *in vivo*^19^.

Despite successes in mice and humans, the induction of PGCLCs from the PSCs of large animals such as pigs is still a great challenge; the porcine model would be ideal for clinical medicine applications owing to similarities with human physiology and metabolism. The differentiation of germ cells from porcine PSCs may result in many applications, such as animal disease modeling, animal breeding and the conservation of excellent breeds.

In this study, we developed a method to induce piPSCs into GCLCs *in vitro* and made use of an *in vivo* niche, i.e., mouse seminiferous tubules, to test the development potential of these cells. PGCLCs were induced from piPSCs and further differentiated into SSCLCs. Interestingly, the induced PGCLCs proliferated and developed for more than 6 weeks *in vivo* and exhibited germ cell features after injection into the seminiferous tubules of immunodeficient mice that lacked endogenous germ cells.

## Results

### Pluripotency and differentiation potential of piPSCs

**Figure 1:**
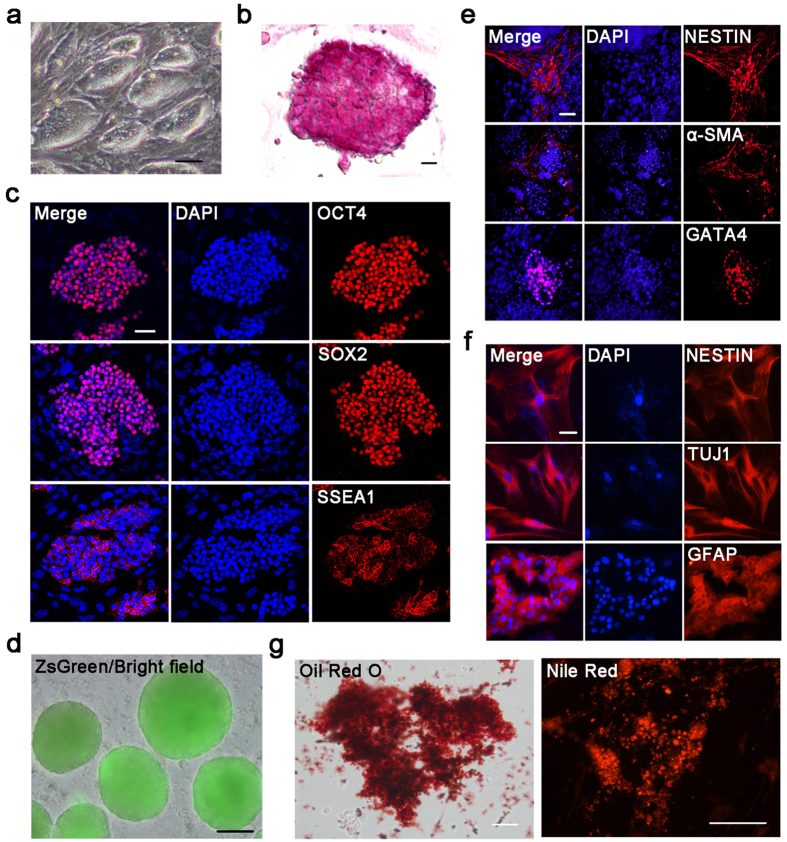
Pluripotency and differentiation potential of porcine iPSCs. (**a**) The piPSC colonies. Scale bar, 100 μm. (**b**) AP staining of iPSCs. Scale bar, 10 μm. (**c**) Immunofluorescence staining of pluripotent markers in iPSCs. Nuclei were stained with DAPI (Blue). Scale bar, 50 μm. (**d**) *In vitro* embryoid body formation. Scale bar, 100 μm. (**e**) Immunofluorescence staining of three germ layers markers after *in vitro* differentiation of iPSCs. Nuclei were stained with DAPI (Blue). Scale bar, 50 μm. (**f**) Characterization of iPSC-derived nerve cells by immunofluorescence staining. Expression of the cell type-specific markers, NESTIN, TUJ1 and GFAP in neural progenitor cells, neurons, or astrocytes, respectively. Nuclei were stained with DAPI (Blue). Scale bar, 25 μm. (**g**) Adipogenic differentiated cells stained with oil red O and nile red, respectively. Scale bar, 50 μm.

To facilitate *in vivo* tracking of piPSC-derived cells, we generated the piPSCs from porcine embryonic fibroblasts containing ZsGreen, a bright green fluorescent protein. The iPSCs were maintained in 2i plus LIF medium^20^. Similar to mouse PSCs, piPSCs showed a compact and dome-like colony morphology (Fig. 1a). These cells were also alkaline phosphatase (AP)-positive and expressed pluripotency markers, such as OCT4/POU5F1, SOX2 and SSEA1 (Fig. 1b,c).

We next investigated the multilineage differentiation potential of the piPSCs by an embryoid body (EB) assay (Fig. 1d). The results indicated that the piPSCs could differentiate into three germ layers, including endoderm (GATA4), mesoderm (α-SMA) and ectoderm (NESTIN) (Fig. 1e). To examine further the differentiation potential of the piPSCs, we cultured piPSCs in adipogenesis or neurogenesis induction media for 1–2 weeks to promote the directed differentiation of the two types of cells. In the adipogenic differentiation group, the induced cells showed an accumulation of intracellular lipid droplets detected by Oil red O and Nile red staining (Fig. 1f). After approximately 15 days of culture in the neurogenesis condition, piPSCs had differentiated into NESTIN^+^ neural progenitor cells, TUJ1^+^ neurons, or GFAP^+^ astrocytes, which were detected by immunofluorescence staining (Fig. 1g). These results indicated that the piPSCs have the potential for multilineage differentiation. Thus, we used these cell lines for the direct induction of germ cells.

### *In vitro* induction of PGCLCs from piPSCs via an epiblast-like state

**Figure 2:**
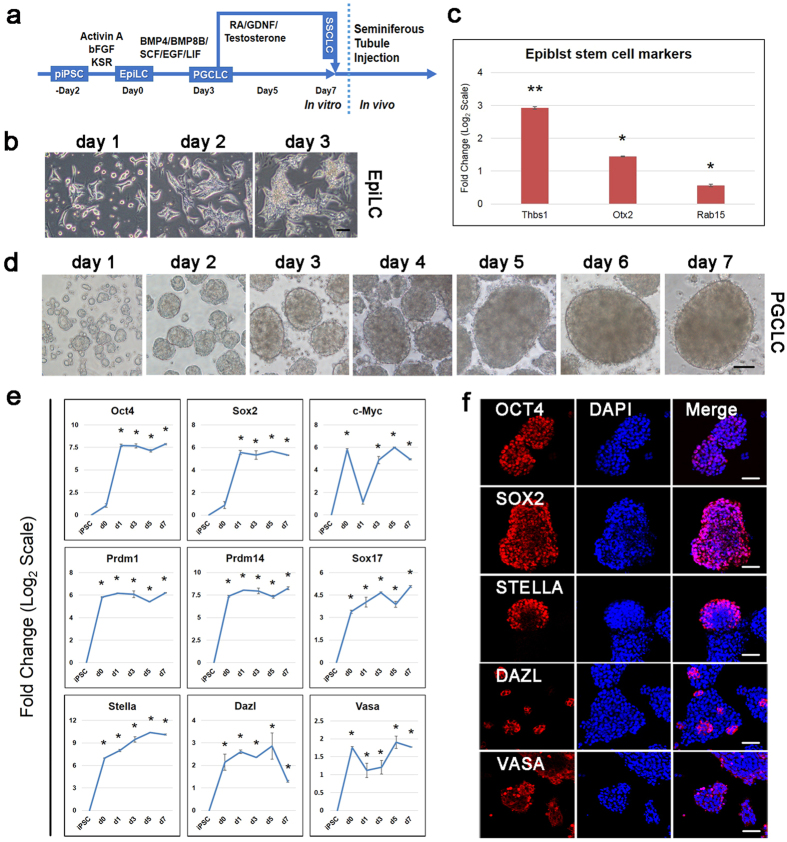
Induction of PGCLCs from porcine iPSCs. (**a**) Schematic of the procedure used to differentiate piPSCs toward male germ cells. (**b**) Morphologies of EpilCs induced from iPSCs. Scale bar, 50 μm. (**c**) Gene expression dynamics during EpiLC induciton by quantitative RT-PCR. The piPSCs were used as the control. Error bars indicate SDs from two independent experiments. *0.001 ≤ p ≤ 0.05; **p < 0.001. (**d**) Development of day 1–7 PGCLCs induced from EpiLCs. Scale bar, 50 μm. (**e**) Gene expression dynamics during PGCLCs induction by quantitative RT-PCR. The piPSCs were used as the control. The d0, d1, d3, d5 and d7 are short for the day 0, day 1, day 3, day 5 and day 7 during induction of PGCLCs from EpiLCs, respectively. Error bars indicate SDs from two independent experiments. *0.001 ≤ p ≤ 0.05. (**f**) Immunofluorescence staining of PGC markers after *in vitro* differentiation. Scale bar, 50 μm.

Previous reports have shown that mouse PSCs were able to convert into PGCLCs via an epiblast-like state^1^,^21^, in addition, human PSCs have been pre-differentiated toward PGC precursors^13^,^14^. After optimization of the induction systems, we developed a procedure for porcine PGCLC differentiation from piPSCs (Fig. 2a). To induce epiblast stem cell-like cell (EpiLC) differentiation, piPSCs were cultured for 2 days in medium containing Activin A, bFGF and 1% knockout serum replacement (KSR) (Fig. 2b). After 2 days of culture, the expression of *THBS*, *OTX2* and *RAB15*, markers of epiblast stem cells, were significantly upregulated (Fig. 2c). These cells were disaggregated and cultured in medium supplemented with bone morphogenetic protein 4 (BMP4), bone morphogenetic protein 8a (BMP8a), leukemia inhibitory factor (LIF), stem cell factor (SCF), and epidermal growth factor (EGF) for further differentiation into PGCLCs (Fig. 2d). Similar to mouse and human cells, porcine PGCLCs proliferated robustly, forming tight and large cell aggregates in the first 4 days; however, the proliferation rate decreased after 5 days under the PGCLC induction conditions (Fig. 2d).

Subsequently, we analyzed the gene expression dynamics during 7 days of PGCLC induction using quantitative RT-PCR (Fig. 2e). Pluripotent marker genes *OCT4* and *SOX2* exhibited a modest upregulation, while *C-MYC* was downregulated slightly on days 1/3/5/7. High expression of *SOX17* is typical of human induced PGCLCs^12^,^13^. In our work, we found upregulation of the endodermal factor *SOX17* during differentiation. The key genes for PGC specification and development were also upregulated during PGCLC formation, including *PRDM1*, *PRDM14*, and *STELLA*. In particular, *STELLA* was significantly elevated on days 1/3/5 and was downregulated thereafter. Genes associated with later germ cell development, such as *DAZL* and *VASA*, showed only a moderate upregulation. STELLA, DAZL and VASA proteins could be detectable in PGCLCs, but not in the iPSCs by immunofluorescence staining (Fig. 2f and Supplementary Fig. 1a). The procedure yielded more STELLA-positive cells, whereas only a small number of DAZL- and VASA-positive cells were present in the whole PGCLC population (Fig. 2f). Both OCT4 and SOX2 were highly expressed in a mass of PGCLCs (Fig. 2f), demonstrating the pluripotency of the induced cells. These results indicate that the method we used is feasible for the efficient and rapid differentiation of piPSCs into germ cell lineages.

### Epigenetic status of PGCLCs

**Figure 3:**
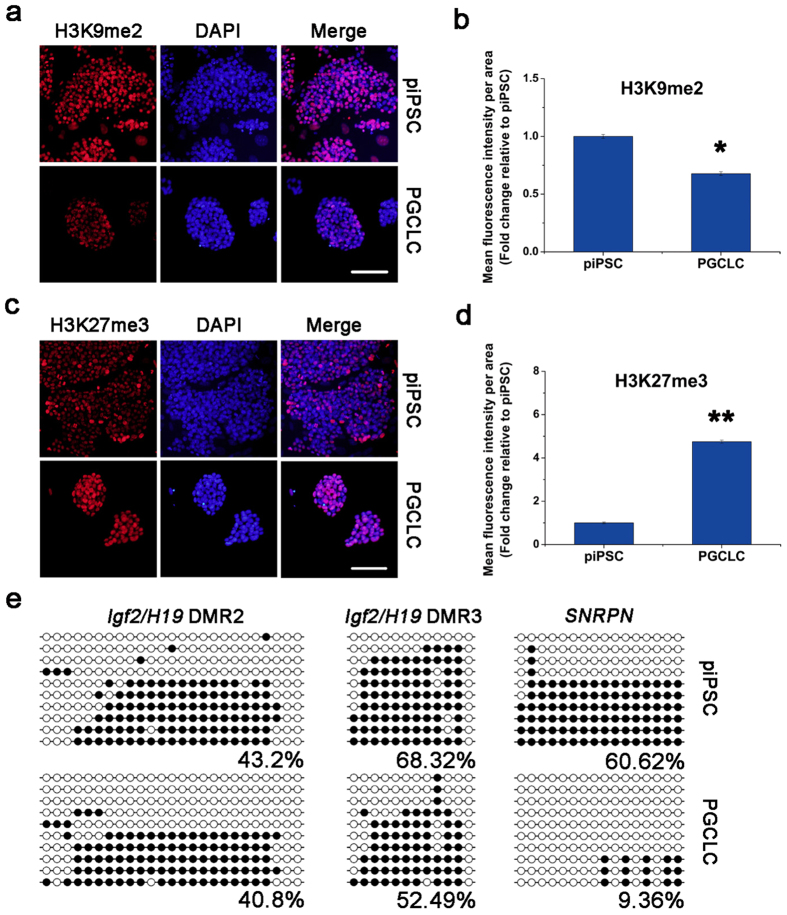
Epigenetic properties of the PGCLCs. (**a,c**) Immunofluorescence analyses of H3K9me2 (**a**) and H3K27me3 (**c**) in PGCLCs under the induction system. Nuclei were stained with DAPI (Blue). Scale bar, 100 μm. (**b,d**) Quantification of relative H3K9me2 (**b**) and H3K27me3 (**d**) fluorescence intensity using ImageJ software. Fluorescence in iPSCs is as a standard. *p ≤ 0.05; **p ≤ 0.001. (**e**) Bisulfite sequence analysis of DMRs of paternally and maternally imprinted genes in iPSCs (top) and the PGCLCs (bottom). White and black circles represent unmethylated and methylated CpGs sequences, respectively.

During germ cell development, PGCs undergo dynamic epigenetic reprogramming events, including global DNA demethylation and histone modifications, H3K27me3 and H3K9me2^22^,^23^,^24^,^25^,^26^. Therefore, we assessed the epigenetic properties of induced PGCLCs. Compared to piPSCs, PGCLCs exhibited reduced H3K9me2 levels and elevated H3K27me3 levels via immunofluorescence staining (Fig. 3a–d). The dynamics of histone methylation changes in the PGCLCs was in good agreement with those of the porcine PGCs after 15 days post-coitum (dpc)^22^. Furthermore, the profiles of paternal (*IGF2/H19*) and maternal (*SNRPN*) imprinting genes were also examined during PGCLC formation. Both *IGF2/H19* and *SNRPN* became demethylated in the PGCLCs (Fig. 3e), suggesting that the PGCLCs may have initiated the imprint erasure process. These findings are in agreement with those that were observed in migrating and gonadal porcine PGCs *in vivo*, which showed major DNA demethylation after colonizing the gonadal ridges^27^.

### Transcriptome analysis of porcine PGCLCs

To characterize comprehensively the induced PGCLCs, we analyzed transcriptomes of the induced PGCLCs by RNA sequencing (RNA-Seq) and compared them with published data of human^14^ and mouse^1^ PGCLCs.

**Figure 4:**
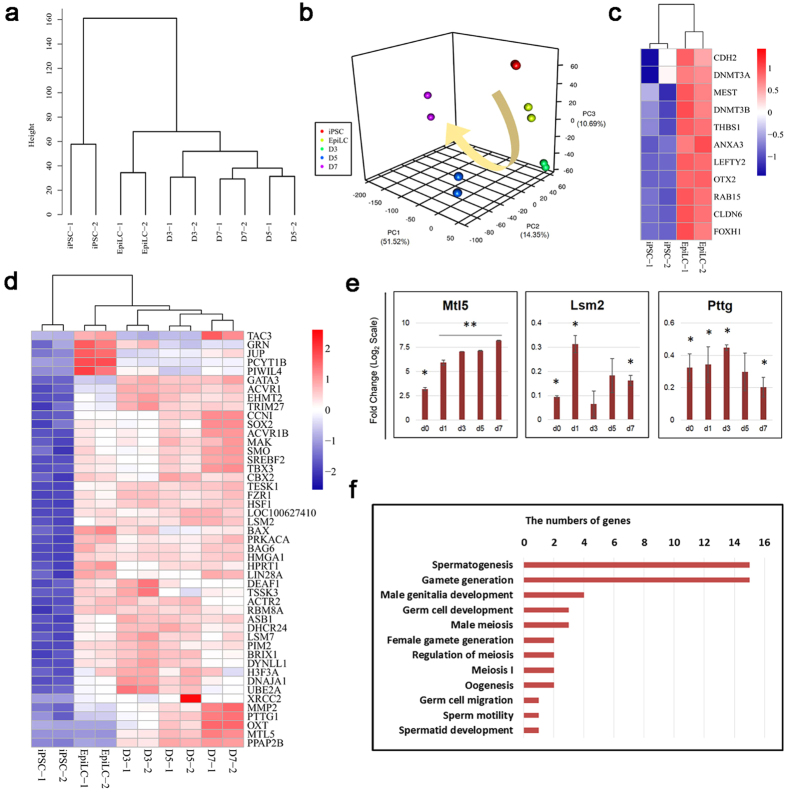
Transcriptional profiles during PGCLC induction. (**a**) Unsupervised hierarchical clustering (UHC) of gene expression in iPSCs, EpiLCs and PGCLCs. (**b**) PCA of RNA-seq data. Arrowline indicates potential progression from iPSCs to PGCLCs. (**c**) Heatmap of the expression of marker genes of epiblast stem cells in iPSCs and EpiLCs. (**d**) Heatmap of the expression of selected genes associated with germ cell development. (**e**) Quantitative RT-PCR analyzed the expression of genes selected by heatmap. The piPSCs were used as the control. The d0, d1, d3, d5 and d7 are short for the day 0, day 1, day 3, day 5 and day 7 during induction of PGCLCs from EpiLCs, respectively. Error bars indicate SDs from two independent experiments. *0.001 ≤ p ≤ 0.05; **p < 0.001. (**f**) GO terms associated with germ cell developement of upregulated porcine day 7 PGCLCs compared with iPSCs.

Unsupervised hierarchical clustering (UHC) analysis classified these cells into two large clusters, one being iPSCs and the other being EpiLCs and day 3/5/7 PGCLCs (Fig. 4a). Consistently, principal component analysis (PCA) revealed clear differences between iPSCs, EpiLCs and PGCLCs, as well as a processive and directional transition during induction (Fig. 4b). These data indicate that PGCLCs and iPSCs have distinct global gene expression profiles.

During EpiLC differentiation from iPSCs, expression levels of genes associated with the primed state were elevated (Fig. 4c). These results were consistent with the quantitative RT-PCR data (Fig. 2c). The heat map illustrates that EpiLCs share properties analogous to those of epiblast stem cells. A heat map of mRNA expression during PGCLC induction revealed that day 3/5/7 PGCLCs shared expression of genes related to “germ cell development and migration,” genes for spermatogenesis and meiosis, and genes associated with gamete generation (Fig. 4d). Once cells had differentiated, some gamete generation-related genes (i.e., *TRIM27*, *TSSK3*, *DHCR24*, *DNAJA1* and *BRIX1*) and other genes (i.e., *ACTR2*, *PIM2*, and *UBE2A*), which have been shown to play roles in mouse or human meiosis^28^,^29^,^30^, were highly expressed on days 3/5 (Fig. 4d). Additionally, with more induction days, higher “gamete generation- and spermatogenesis-” related gene expression (*CCNI*, *ACVR1B*, *MAK*, *MTL5*, *SMO*, *SREBF2* and *PTTG1*) and “germ cell development” gene expression (*LSM2* and *PPAP2B*) were observed (Fig. 4d). Quantitative RT-PCR corroborated these data (Fig. 4e).

Gene ontology (GO) statistical enrichment analysis of differentially expressed genes (DEGs) between day 7 PGCLCs and iPSCs revealed that the most enriched genes were those associated with spermatogenesis and gamete generation during germ cell induction (Fig. 4f). We further assessed DEGs between human/mouse PGCLCs and iPSCs/ESCs using human RNA-Seq data from Sasaki *et al*.^14^ and mouse microarray data from Hayashi *et al*.^1^. The analysis of porcine GO terms was consistent with that of human and mouse terms (Supplementary Fig. 2).

Taken together, these induced cells exhibited a gene expression pattern similar to that of germ cells. In addition, the porcine PGCLCs we generated were transcriptionally indistinguishable from the human and mouse counterparts. These analyses show a clear description of main transcriptional properties of two cell types during *in vitro* PGCLC specification.

### Differentiation of PGCLCs into SSCLCs

**Figure 5:**
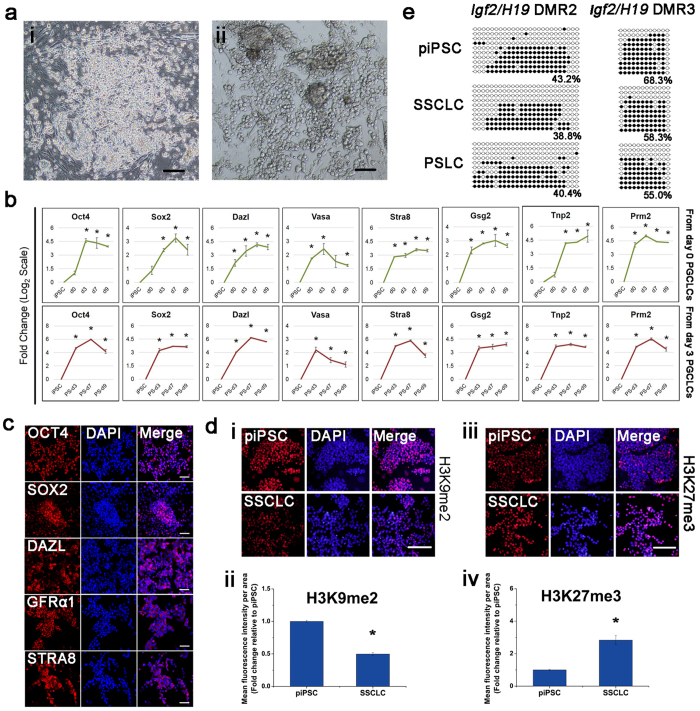
Characterization of the SSCLCs. (**a**) Morphologies of SSCLCs. Scale bar, 200 μm (i,ii). (**b**) Gene expression profiles during SSCLCs induction from day 0 PGCLCs (top) and day 3 PGCLCs (bottom) by quantitative RT-PCR. The piPSCs were used as the control. The d3, d7 and d9 are short for the day 3, day 7 and day 9 during induction of SSCLCs from EpiLCs (top) and PGCLCs (bottom), respectively. Error bars indicate SDs from two independent experiments. *0.001 ≤ p ≤ 0.05. (**c**) Immunofluorescence staining of SSC markers after *in vitro* differentiation. (**d**) Immunofluorescence analyses of H3K9me2 (i) and H3K27me3 (iii) in SSCLCs. (i,iii) Immunostaining of proteins. Nuclei were stained with DAPI (Blue). DAPI staining, in the middle. Merged images, on the right. Scale bar, 100 μm. (ii,iv) Quantification of relative H3K9me2 (ii) and H3K27me3 (iv) fluorescence intensity using ImageJ software. Fluorescence in iPSCs is as a standard. *p ≤ 0.05. (**e**) Bisulfite sequence analysis of DMRs of imprinted genes in iPSCs (top) and SSCLCs (bottom). White and black circles represent unmethylated and methylated CpGs sequences, respectively. SSCLC indicates SSCLCs from day 0 PGCLCs, and PSLC is short for SSCLCs from day 3 PGCLCs.

Spermatogonial stem cells (SSCs) provide the foundation for spermatogenesis^31^. We further induced PGCLCs into SSCLCs, because the spermatogenic lineage has shown an excellent ability to colonize testes and restore fertility in mice and non-human primates (NHPs)^32^,^33^. Based on the above observations, we cultured day 2 EpiLCs (i.e., day 0 PGCLCs) and PGCLCs on different days in RGT medium to transform them to the SSC-like morphology. We observed that SSC-like clumps emerged and expanded, forming SSC-like colonies from day 0 PGCLCs and day 3 PGCLCs after 3 days (Fig. 4a and Supplementary Fig. 3a,b). Quantitative RT-PCR results revealed that germ cell marker *DAZL*, but not *VASA*, was elevated, and SSC marker *STRA8* was also elevated once large masses of cells formed (Fig. 5b), as shown by identification of the expression of DAZL, GFRα1 and STRA8 proteins (Fig. 5c). Haploid markers *GSG2*, *TNP2* and *PRM2* were elevated. Flow cytometry analysis of DNA content showed the presence of haploid in induced SSCLCs and PSLCs (1.23% and 3.22%, respectively). As a negative control, 0.31% of haploid cells were detectable in the piPSCs (Supplementary Fig. 3c). These data indicate that meiosis has initiated since SSCLCs were induced. Compared with SSCLCs from day 0 PGCLCs, SSCLCs from day 3 PGCLCs showed higher gene expression of germ cell markers, including *DAZL*, *STRA8*, *GSG2, TNP2* and *PRM2* (Fig. 5b). Additionally, we detected that pluripotent markers *OCT4* and *SOX2* were expressed at relatively high levels and so were the corresponding proteins (Fig. 5c). These marker genes were also examined in the SSCs isolated from pig testes by quantitative RT-PCR (Supplementary Fig. 3d), indicating that the germ cell marker gene expression dynamics in the induced SSCLCs are similar to those in SSCs from pig testes. These findings demonstrate that SSCLCs have pluripotency and properties similar with SSCs in testis, and parts of these cells can enter meiosis.

During spermatogenesis, the re-acquisition of H19 methylation has been reported^34^,^35^. In the process, H3K27me3 was retained^36^, and the H3K9me2 level was low in spermatocytes and then high in spermatogonia and spermatids^37^. We evaluated the expression levels of histone methylation and the imprinting states of DMR2/3 of the *IGF2/H19* gene cluster. The expression levels of H3K9me2 and H3K27me3 changed because of the induction of SSCLCs; a lower expression level of H3K9me2 and a higher expression level of H3K27me3 were observed (Fig. 5d). Notably, *IGF2/H19* gene cluster DMR2 and DMR3 exhibited a reduced level of methylation compared with that of iPSCs; in contrast, a slightly higher level of methylation was observed compared with that of PGCLCs (Fig. 5e). These findings suggest that the *in vitro* culture conditions we used closely mirror the events of *in vivo* spermatogenesis, and facilitate the generation of SSCLCs.

### Xenotransplantation directs GCLC survival, proliferation and further development *in vivo*

**Figure 6:**
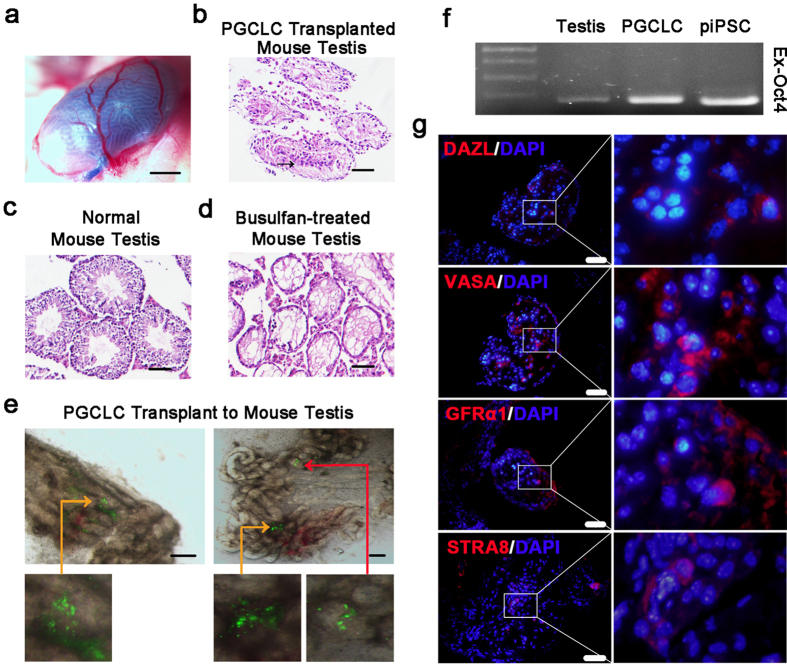
Transplantation of PGCLCs into busulfan-treated mouse testes. (**a**) A testis with successful injection into the seminiferous tubules. Scale bar, 1 mm. (**b**) HE staining of testes after over 6 weeks post-transplantation of PGCLCs. The black arrow indicates the individual tubule. Scale bar, 50 μm. (**c**) HE staining of normal mouse testes before busulfan treatment. Scale bar, 50 μm. (**d**) HE staining of busulfan-treated germ cell depletion testes. Scale bar, 50 μm. (**e**) The seminiferous tubules transplanted with the PGCLCs. The red arrow indicates donor-derived chains of the tubules. Orange arrows indicate donor-derived clusters of the tubules. Scale bar, 500 μm. (**f**) Detection of donor-derived cells in testes by PCR. (**g**) Immunohistochemical analysis of testis xenografts from induced PGCLCs. Cross sections immunostained for DAZL, VASA, GFRα1 and STRA8. Nuclei were stained with DAPI (Blue). Scale bar, 50 μm.

The germ cell transplantation assay provides a platform for donor-derived cells to colonize, complete spermatogenesis and produce sperm *in vivo*. To evaluate the induced GCLC developmental ability, we xenotransplanted PGCLCs into the lumen of mouse seminiferous tubules lacking endogenous male germ cells (Fig. 6a). ZsGreen-positive donor cells engrafted both near the basement membrane and in the tubule lumen. Parts of the seminiferous tubules were colonized with PGCLCs, which can survive for over 6 weeks and form chains and clusters in the mouse seminiferous tubules after transplantation owing to instructive cues from the *in vivo* niche (Fig. 6e). To detect further whether ZsGreen-positive cells are donor cells, we amplified the exogenous *OCT4* gene by PCR. PCR analysis of DNA extracted from testes containing ZsGreen-positive cells revealed the presence of the donor cells (Fig. 5f). The hematoxylin-eosin (HE) staining results showed the differences among normal, busulfan-treated and donor cell-transplanted testes. Normal testes contained spermatogonia and mature sperm, while busulfan-treated testes only had empty lumens eliminating endogenous germ cells (Fig. 5c,d). Testes transplanted with PGCLCs were similar to normal testes (Fig. 5b), suggesting that donor cells survived and developed when exposed to the mouse seminiferous tubules for over 6 weeks. For immunohistochemistry (IHC) of serial cross-sections of fixed tissue, we chose some well-known germ cell markers, including DAZL, VASA, GFRα1 and STRA8^19^,^21^,^38^. Staining of serial sections from a 10-week-old normal mouse testis revealed that germ cells that expressed these markers existed either near the basement membrane or in the seminiferous tubule lumen (Supplementary Fig. 5a). In contrast, negative immunostaining results were observed in busulfan-treated testes (Supplementary Fig. 5b). Based on the IHC assay of PGCLC-transplanted testis sections, a portion of the tubules contained DAZL-positive germ cells or more VASA-positive germ cells (Fig. 6g). In some tubules, donor cells were also identified by the positive immunostaining for SSC markers, GFRα1 and STRA8 (Fig. 6g).

In addition to the xenotransplantation of PGCLCs, we also transplanted SSCLCs into seminiferous tubules to examine what developmental stage they could reach. As shown in Supplementary Fig. 4b, seminiferous tubules, which were different from those of busulfan-treated testes visualized by HE staining, contained small chains and large clusters of ZsGreen-positive cells (Supplementary Fig. 4a). Moreover, germ cell markers were identified by immunofluorescence staining of DAZL, VASA, GFRα1 and STRA8 (Supplementary Fig. 4c). After GCLCs transplantation, tubules without ZsGreen cells were also isolated and stained. The results showed the empty lumen and negative marker proteins staining (Supplementary Fig. 6). Taken together, these results indicate that induced porcine PGCLCs and SSCLCs acquire male germ cell developmental potential *in vivo* and present germ cell characteristics.

## Discussion

In the present study, we present a defined differentiation method via a combination of *in vitro* priming and an *in vivo* niche to complete germ cell induction using piPSCs as starting cells. The experimental conditions, being different from those reported previously, allowed the induction of either PGCLCs or SSCLCs in response to different factors in an efficient way. Mouse PGCLCs are induced via an EpiLC state^1^, while human PGCLCs are derived through a mesoderm-like cell population^13^,^14^. We cultured porcine PSCs, which resembled mouse ESCs, first in the EpiLC medium for 2 days and then in the PGCLC medium; these steps were followed by differentiation of the SSCLCs (Fig. 2a). BMP4 played a vital role in the specification of porcine PGCLCs in our study, in accordance with studies of inducing mouse and human PGCLCs^1^,^5^,^12^,^13^. In addition to BMPs, both EGF and SCF contribute to enhancing the growth of mouse PGCs^2^. We observed that aggregates of porcine PGCLCs grew and expanded upon exposure to EGF, SCF and BMPs. Upon PGCLC induction, a majority of cells acquired *PRDM1*, *PRDM14* and *STELLA* at sufficient levels and progressed toward PGCLCs, as shown by the morphological, genetic and epigenetic profiles (Figs 2 and 3). The genes for spermatogenesis and gamete generation were enriched, which is similar to findings in mice and humans^1^,^12^,^13^,^14^ (Fig. 4 and Supplementary Fig. 2). The upregulation of *SOX17* was consistent with that in human PGCLC induction *in vitro* (Fig. 2e)^4^,^13^. The porcine PGCLCs showed early signs of histone methylation and DNA demethylation (Fig. 3a–e), which were remarkably similar to those of germline epigenetic reprogramming^23^. Taken together, our findings suggest that BMPs, EGF and SCF play positive roles in the induction of PGCLCs from porcine PSCs.

The differentiation of PSCs into SSCLCs is necessary for evaluating the possibility of restoring fertility. To establish differentiation systems that mimic the *in vivo* niche, cytokines and hormones, which promote SSC development and initiate meiosis, were added. Glial cell line-derived neurotrophic factor (GDNF) is required for SSC maintenance and proliferation^39^. RA stimulates PGC division and contributes to meiosis through the CYP26/STRA8 signal pathways^7^,^40^,^41^. Testosterone secreted by Leydig cells is necessary for spermatogenesis, and it also acts on Sertoli cells to promote male germ cell differentiation^42^. During SSCLC induction, we observed SSC-like morphology and the expression of meiosis markers that are similar to those in SSCs from pig testes, including *STRA8*, *GSG2, TNP2 and PRM2*. Besides, some haploid cells were produced during induction (Supplementary Fig. 3c). In contrast with published papers about mice and humans^5^,^6^,^10^,^18^, our work outlines a procedure for generating large masses of porcine SSCLCs that express positive SSC and haploid markers and have epigenetic patterns (Fig. 5). This work also represents a direct and rapid method for differentiating piPSCs into germ cell lineages using extrinsic cytokines and hormones without any genetic manipulation. The induction of EpiLCs without going through PGC differentiation is an important strategy for decreasing induction time and thereby increasing the potential for applications.

Xenogeneic germ cell transplantation to rodent testes cannot result in complete spermatogenesis because of anatomical and species differences^43^,^44^, but rodent seminiferous tubules indeed represent a promising recipient microenvironment for male germ cell survival and development. The culture system we used attempts to generate male germ cells of different stages by mimicking the *in vivo* niche and providing the cytokines and hormones mainly secreted by other types of cells. Based on our observations, porcine induced GCLCs have the potential to be directed into the germ cell fate *in vivo*. First, donor cells in the formation of chains and clusters resided in the basal membrane or the center of the mouse seminiferous tubules (Fig. 6e and Supplementary Fig. 4a). Second, donor cells in the testes expressed key germline markers, including DAZL, VASA, GFRα1 and STRA8 (Fig. 6g and Supplementary Fig. 4c). Therefore, these results demonstrate that the induction of germ cells from porcine PSCs both *in vitro* and *in vivo* is feasible; however, apart from mice, recipient testes from the same species should be considered for promoting *in vivo* differentiation because livestock-derived donor cells cannot complete spermatogenesis in rodent testes^43^,^44^. Some roadblocks, e.g., no suitable pig recipients for testing *in vivo* development of induced PGCLCs, need to be overcome to achieve the goal of accomplishing germ cell meiosis and development to more mature stages.

The mechanism for PGC specification and SSC development in pigs remains unclear. The culture conditions used in the present study allow the derivation of PGCLCs and SSCLCs in a relatively efficient way and thus may lay the foundation for elucidating a development mechanism because of the material limitations *in vivo*. Continued research will provide a more comprehensive understanding of porcine male germ cell induction and provide better ways of differentiating PSCs into GCLCs for reproductive medicine and animal breeding.

## Materials and Methods

### Animals

Immunodeficient Nu/Nu mice were purchased from Vital River Laboratories (Beijing, China) and used for the xenotransplantation assay. Animal care was in accordance with the guidelines of China Agricultural University for animal welfare. All animal experiments in the present study were approved by the Animal Care and Use Committee of China Agricultural University.

### Cell culture and Differentiation *in vitro*

The piPSCs were generated from the pig embryonic fibroblasts containing ZsGreen and were maintained on mitomycin-treated mouse embryonic fibroblasts (called feeder cells) in DMEM supplemented with 15% ESC fetal bovine serum (FBS) (Gibco), nonessential amino acids (NEAA), L-glutamine, penicillin/streptomycin (all from Gibco), β-mercaptoethanol (Sigma), and human LIF and 2i (CHIR99021 and PD0325901) (Selleck) (called 2i plus LIF medium). The medium was changed every day, and cells were passaged every 2–3 days by TrypLE (Invitrogen) dissociation of the culture into single cells.

For the formation of embryoid bodies (EBs), piPSCs were digested and collected in the piPSC medium mentioned above removal of human LIF and 2i on a shaker (40 r/min) in a CO_2_ incubator.

To induce differentiation into EpiLCs, piPSCs were dissociated into single cells and plated in gelatin-coated wells of 6-well plates (4 × 10^5^ cells/well) in N2B27 medium including activin A (20 ng/ml, PeproTech), bFGF (12 ng/ml, Invitrogen) and 1% KSR (Gibco). The medium was changed every day.

For PGCLC differentiation, EpiLCs were digested into single cells and then treated with BMP4 (50 ng/ml, PeproTech), BMP8B (50 ng/ml, R&D), SCF (50 ng/ml, PeproTech), EGF (50 ng/ml, PeproTech), and LIF (1000 U/ml, Gibco) in serum-free medium (GMEM with 15% KSR, NEAA, L-glutamine, penicillin/streptomycin, and β-mercaptoethanol).

To differentiate further into SSCLCs, PGCLCs or EpiLCs were digested and plated in gelatin-coated wells of 12-well plates (2 ×10^5^ cells/well) with DMEM medium containing 15% ESC FBS, NEAA, L-glutamine, penicillin/streptomycin, β-mercaptoethanol, and cytokines, including 2 μM RA and 4 ng/ml GDNF, and 1 μM testosterone (called RGT medium). The medium was changed every day.

To induce piPSCs into the adipogenic and neural lineages, cells were cultured in the differentiation medium as reported previously^45^. The adipogenic differentiation medium contained DMEM, 15% FBS, penicillin/streptomycin, 10 mg/L insulin (Sigma), 0.1 mol/L dexamethasone (Sigma) and 0.5 mM isobutyl-methylxanthine; the neural differentiation medium included DMEM, 15% FBS, β-mercaptoethanol, 1 μM RA and 0.5 mM isobutyl-methylxanthine. The adipogenic-differentiated cells were confirmed by Oil Red O (Sigma) and Nile Red (Life technologies) staining. Cells were fixed with 4% paraformaldehyde (PFA), overlaid with 0.15% Oil Red O dye for 15 min, washed with 60% 2-propanol solution twice and rinsed in distilled water.

### Quantitative RT-PCR and Bisulfite sequencing

Total RNA was extracted using a Qiagen RNeasy mini RNA kit (Qiagen, Hilden, Germany). For quantitative RT-PCR, total RNA from each sample was reverse-transcribed by M-MLV Reverse Transcriptase (Promega), and the cDNAs were used for quantitative RT-PCR analysis with SYBR Green (Roche). The data were analyzed using the Log_2_ Scale method. The ΔCT was calculated using GAPDH as an internal control. The primers used are listed in Table 1.

Genomic DNA was isolated with a DNeasy Blood and Tissue kit (Qiagen) and then were prepared for bisulfite reactions with an EZ DNA Methylation-Gold kit (Zymo Research). PCR amplification of DMRs of imprinted genes was performed using previously reported primers^47^,^48^. The PCR products were cloned into the PMD-19T vector (TAKARA) and were sequenced. Sequences were analyzed using the Quantification tool for Methylation Analysis (QUMA).

### AP staining and Immunofluorescence staining

Cellular AP activity was detected using an AP Detection Kit (Millipore) according to the manufacturer’s instructions. For immunofluorescence staining, cells were fixed with 4% PFA for 30 min, permeabilized with 0.2% Triton X-100 for 15 min, and then blocked with the immunostaining blocking buffer (Beyotime) for 1 h. Primary and secondary antibodies were incubated overnight at 4 °C and for 1 h at room temperature, respectively. Membrane proteins were not permeabilized with 0.2% Triton X-100. Nuclei were stained with 1 μg/ml of DAPI for 5 min at room temperature. The primary antibodies used here were as follows: OCT4 (1:300, sc-5279, Santa Cruz), SOX2 (1:300, ab97959, Abcam), SSEA1 (1:300, ab16285, Abcam), STELLA (1:300, ab19878, Abcam), DAZL (1:300, ab34139, Abcam), VASA (1:300, ab13840, Abcam), STRA8 (1:300 ab49602, Abcam), GFRα1 (1:300, ab84106, Abcam), PRDM1 (1:100, 9115, CST), PRDM14 (1:300, ab28638, Abcam), SOX17 (1:100, 81778, CST), GATA4 (1:300, ab84593, Abcam), NESTIN (1:300, ab6142, Abcam), α-SMA (1:300, ab5694, Abcam), GFAP (1:300, ab4674, Abcam), TUJ1 (1:300, ab18207, Abcam), H3K9me2 (1:500, ab71604, Abcam), and H3K27me3 (1:500, ab6002, Abcam). The secondary antibodies used in our work were as follows: goat anti-rabbit Alexa Fluor 594 (1:500, A11037, Invitrogen), goat anti-mouse Alexa Fluor 594 (1:500, A11032, Invitrogen), goat anti-mouse Alexa Fluor 594 (1:500, A21044, Invitrogen), and goat anti-chicken Alexa Fluor 594 (1:500, A11042, Invitrogen).

### Transplantation assay and Immunohistochemistry

Nu/Nu mice were used as transplantation recipients. At approximately 6 weeks of age, the recipient mice were injected with busulfan (40 mg/kg body weight) to destroy endogenous germ cells. After more than 4 weeks, these recipients were used for the transplantation assay. The aggregates of PGCLCs were dissociated with TrypLE into single cells and suspended with DPBS (Gibco). 7-10 × 10^5^ cells per testis containing 10% trypan blue were transplanted via the efferent duct into seminiferous tubules^49^. After 6 weeks, recipient mouse testes were harvested for histological analysis and immunohistochemical detection.

The seminiferous tubules with ZsGreen-positive cells were isolated from recipient testes using fluorescence microscopy, immediately collected for genome extraction and fixed with 4% PFA for over 24 hours. These tubules were then embedded in paraffin and sectioned into serial cross-sections of an 8-μm thickness. Testis cross-sections were deparaffinized in xylene and then rehydrated a gradient of ethanol. For antigen retrieval, the slides were boiled in antigen retrieval solution (Beyotime) for 15 min. The sections were blocked with an immunostaining blocking buffer (Beyotime) for 1 h and incubated with primary antibodies, including DAZL, VASA, STRA8 and GFRα1, overnight at 4 °C; the sections were then incubated with secondary antibodies at room temperature for 1 hour. The sections were then washed and sealed with Fluoroshield Mounting Medium with DAPI (Abcam). For HE staining, nuclei were stained with the alum hematoxylin and section slides were stained with eosin. The slides were sealed with nail polish.

### RNA-Seq Sample collection and library preparation

Total RNA was extracted from cells with a Qiagen RNeasy mini RNA kit (Qiagen, Hilden, Germany) according to the standard procedures. The cDNA libraries were constructed using a kit from Illumina following the paired-end sample preparation kit protocol. The oligo (dT) magnetic beads were used to enrich the mRNA of each sample, which were purified and cleaved into short fragments (~330 nt) by adding fragmentation buffer prior to cDNA synthesis. In addition, the short fragments were ligated to sequencing adapters. Fragments with suitable sizes (400∼500 nt) were purified by agarose gel electrophoresis and then were selected to be templates for PCR amplification to produce the library and sequence via the Illumina HiSeq 2500 sequencing platform. RNA-seq was performed on two biological replicates for each cell type.

### Bioinformatic analysis of RNA-seq data

We removed sequencing adapters from reads using Trimmomatic (v0.32)^50^. In addition, we remained reads with a length of no less than 32 bp when one or a few bases having a Q-score lower than 20 were deleted. Additionally, reads without mates were excluded. Then, qualified short reads were aligned to the pig genome Scrofa10.2, which was downloaded from the NCBI ftp site by Tophat (v2.0.9)^51^. We used Bowtie2^52^ to index the genome, and the maximum multiple hits was 20. Tolerances were set to allow at most two mismatches for 25-bp segments and zero mismatches at the splice junctions.

To estimate expression levels of genes or transcripts, we used Cufflinks (v2.1.1)^53^, which tracks changes in expression at the level of transcripts and genes with high reliability. The RefSeq v105 gene annotation GTF (general transfer format) file was from NCBI. EdgeR was used to test differentially expressed transcripts and genes. The test transcripts in the result file with statistical significance (p < 0.05, fold change > 1) were believed to be differentially expressed. PCA was performed using the prcomp function in R 3.1.1. Heat maps were created using the pheatmap package in R 3.1.1.

To compare relative gene expression levels in humans and mice, we used the RNA-seq data from Sasaki^14^ (GSE67259). Processed data were downloaded from the GEO database. Quantile normalization and differential expression analysis was performed using the EdgeR package. To enrich DEGs in GO terms, DEGs were split between up- and downregulated DEGs.

The GO terms were taken from the GO.db package in R 3.0.0. The significance of GO terms from the DEGs was calculated using GOstats, which applies the hypergeometric distribution to calculate the enrichment P-value.

## Additional Information

**How to cite this article**: Wang, H. *et al*. Induction of Germ Cell-like Cells from Porcine Induced Pluripotent Stem Cells. *Sci. Rep*. **6**, 27256; doi: 10.1038/srep27256 (2016).

## Supplementary Material

Supplementary Information

## Figures and Tables

**Table 1:** The primers used for quantitative RT-PCR’.

	Forward primer sequence (5′ to 3′)	Reverse primer sequence (5′ to 3′)
*GAPDH*	GTATGATTCCACCCACGGCA	GCCTTCTCCATGGTCGTGAA
*PRDM14*	AGTGGATGCTTCTCTGCTACC	TGCCTTTCTCTCTTGGTTCA
*PRDM1*	CAGTGCCGTGAAGTTTCCA	AAGGATGCCTCTGCCTGAAC
*SOX17*	CGCACGGAGTTTGAACAATA	CAGACGTCGGGGTAGTTACAG
*THBS*	CCTGCGCAGCCTCTGATACC	AATGCGGTTGGAGCCACACA
*OTX2*	GTCGCGCCGCAAACTCCAAA	CCCGTTCCCTCCCAAGCAAT
*RAB15*	CCGCTTCACCGACAACGAGT	CCCTGGGCCCGTCGATAGTA
*MTL5*	AGTTGAAAGGGGGTACACAAATGCT	GGGGAAAGCTGACCCGTTGAC
*LSM2*	GCGGTGCAAGCACAATGCTC	GCTGGCAACTGCACATAGCG
*MMP2*	TATGGAAACGCCGACGGGGA	GGAACGGGAACTTGCAGGGC
*SMO*	CCCATTCACTCCCGCACCAA	CTGCGAGAGCCAGACACGAG
*STELLA*	CCCGCCTTTCAATCTGTCTCC	TCGCCGAACCGTGTATCGAA
*DAZL*	GGGTCGCTTTGCTTATCCGC	TGCAGCAGACATTACTGCGA
*VASA*	CCTGCCCAGGAATGCCATCA	ACTGGCCAACTTGGAGAATGGT
*STRA8*	TGGAGAAGGGAGCAACCCCA	ACCTGCCACTTTGAGGCTGT
*GSG2*	TTGGGGCAGATGGGAAGAGTA	ACTTCTTTCAGGCCGGTTGA
*TNP2*	CGGAGCTCAGGGCGAAAATAC	TCCCGTGTACAAGCTTTACTCAATG
*PRM2*	GACCAGGGCTGCAGACGGA	TACTCAAGATCTCGTGGGCTCCT
*OCT4*^46^	CAAACTGAGGTGCCTGCCCTTC	ATTGAACTTCACCTTCCCTCCAACC
*SOX2*^46^	CATCAACGGTACACTGCCTCTC	ACTCTCCTCCCATTTCCCTCTTT
*C-MYC*^46^	ATCCAAGACCACCACCACTG	GTTCACAGCAACATTCAGGTAGA
*pMXs-Oct4*^20^	GACGGCATCGCAGCTTGGATACAC	GAGAAGGCGAAGTCGGAAG
