# Supplementary material for: Induction of Germ Cell-like Cells from Porcine Induced Pluripotent Stem Cells
Source: Sci Rep. 2016 Jun 6;6:27256. doi: 10.1038/srep27256 (PMC4893677; doi:10.1038/srep27256)
Supplement: Supplementary Information [file srep27256-s1.doc]

**Induction of Germ Cell-like Cells from Porcine Induced Pluripotent Stem Cells**

**Authors:** Hanning Wang^1^, Jinzhu Xiang^1^, Wei Zhang^1^, Junhong Li^1^, Qingqing Wei^1^, Liang Zhong^1^, Hongsheng Ouyang^2^, Jianyong Han^1^*

**Author affiliations:**

^1^ State Key Laboratories for Agrobiotechnology, College of Biological Sciences, China Agricultural University, Beijing, 100193, China.

^2^ Jilin Provincial Key Laboratory of Animal Embryo Engineering, College of Animal Sciences, Jilin University, 5333 Xi'an Road, Changchun, Jilin, 130062, China.

Correspondence and requests for materials should be addressed to J.H. (email: [hanjy@cau.edu.cn](mailto:hanjy@cau.edu.cn))

**Supplementary Information**

**Supplementary Data**


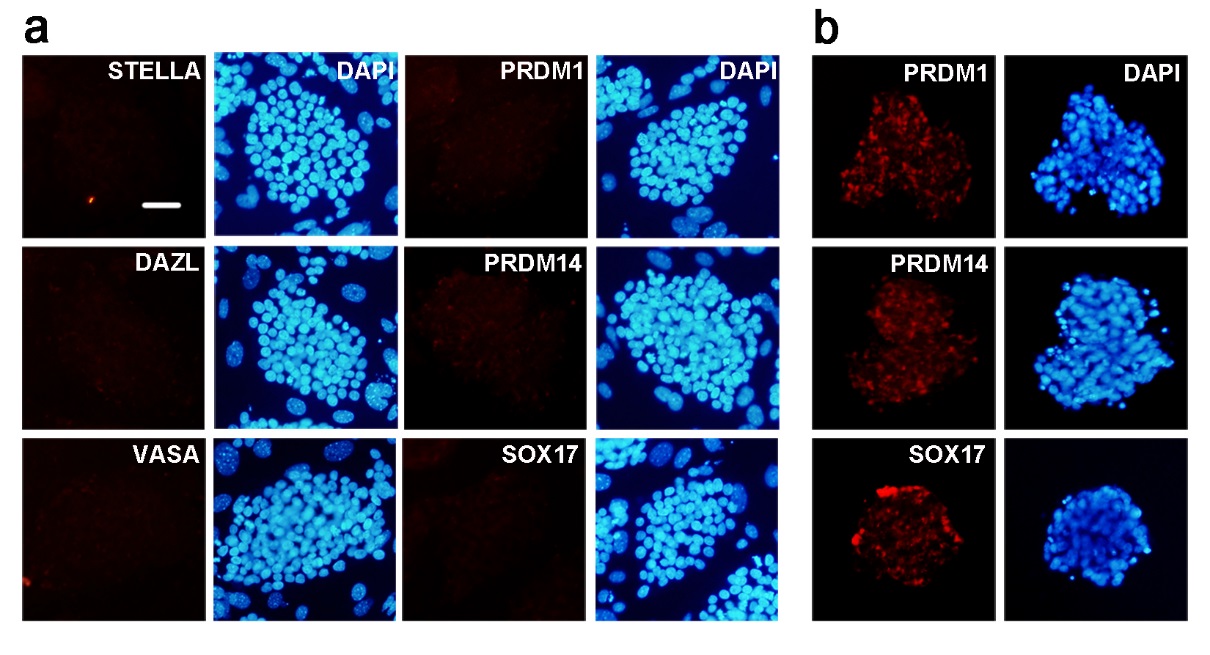


**Supplementary Figure 1. Immunofluorescence staining in iPSCs and PGCLCs**

(a and b) Immunofluorescence staining in iPSCs (a) and PGCLCs (b). Nuclei were stained with DAPI (Blue). Scale bar, 100 μm.


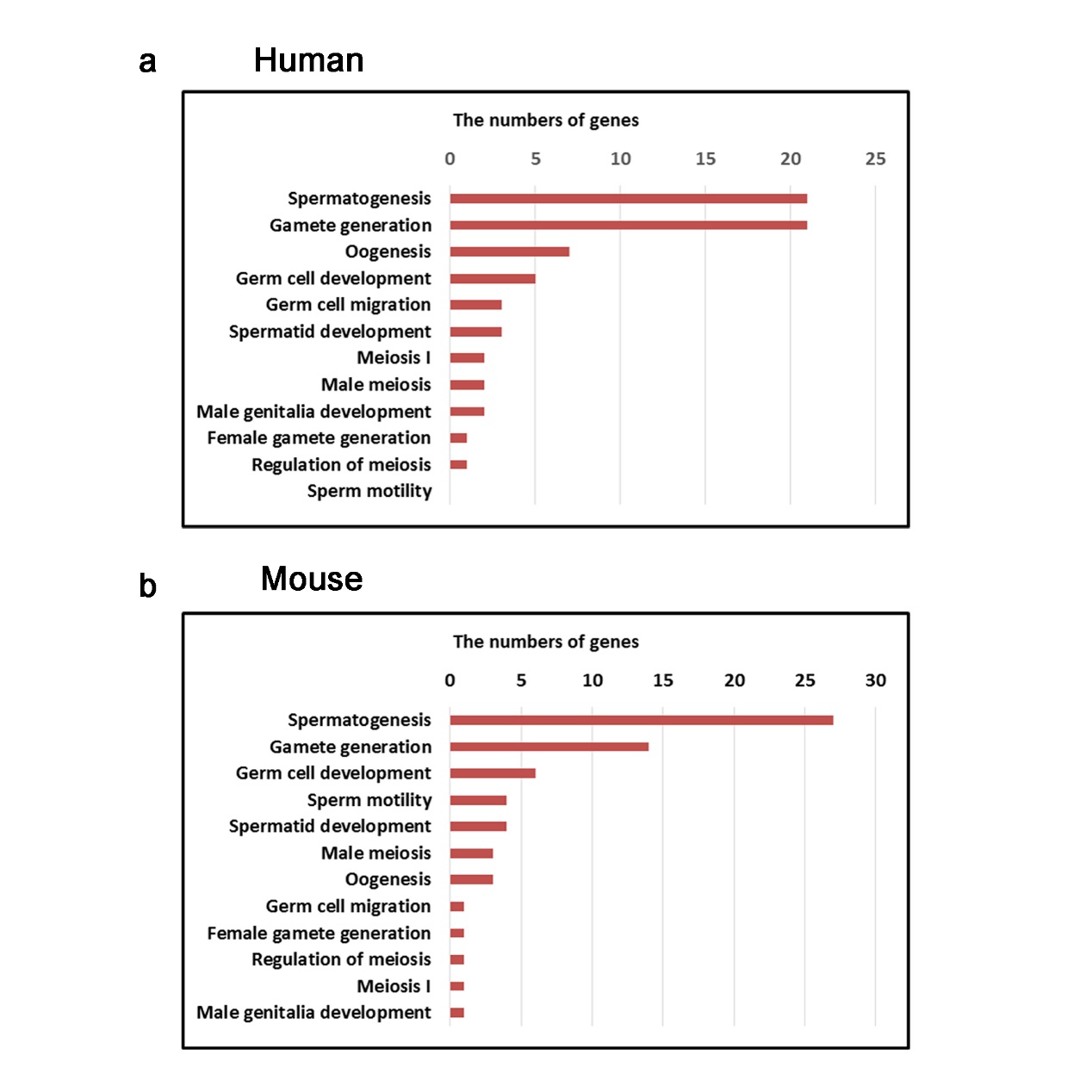


**Supplementary Figure 2. Bar chart of GO terms**

(a) GO analysis associated with germ cell development of upregulated human day 6 PGCLCs compared with iPSCs.

(b) GO analysis associated with germ cell development of upregulated mouse day 6 PGCLCs compared with ESCs.


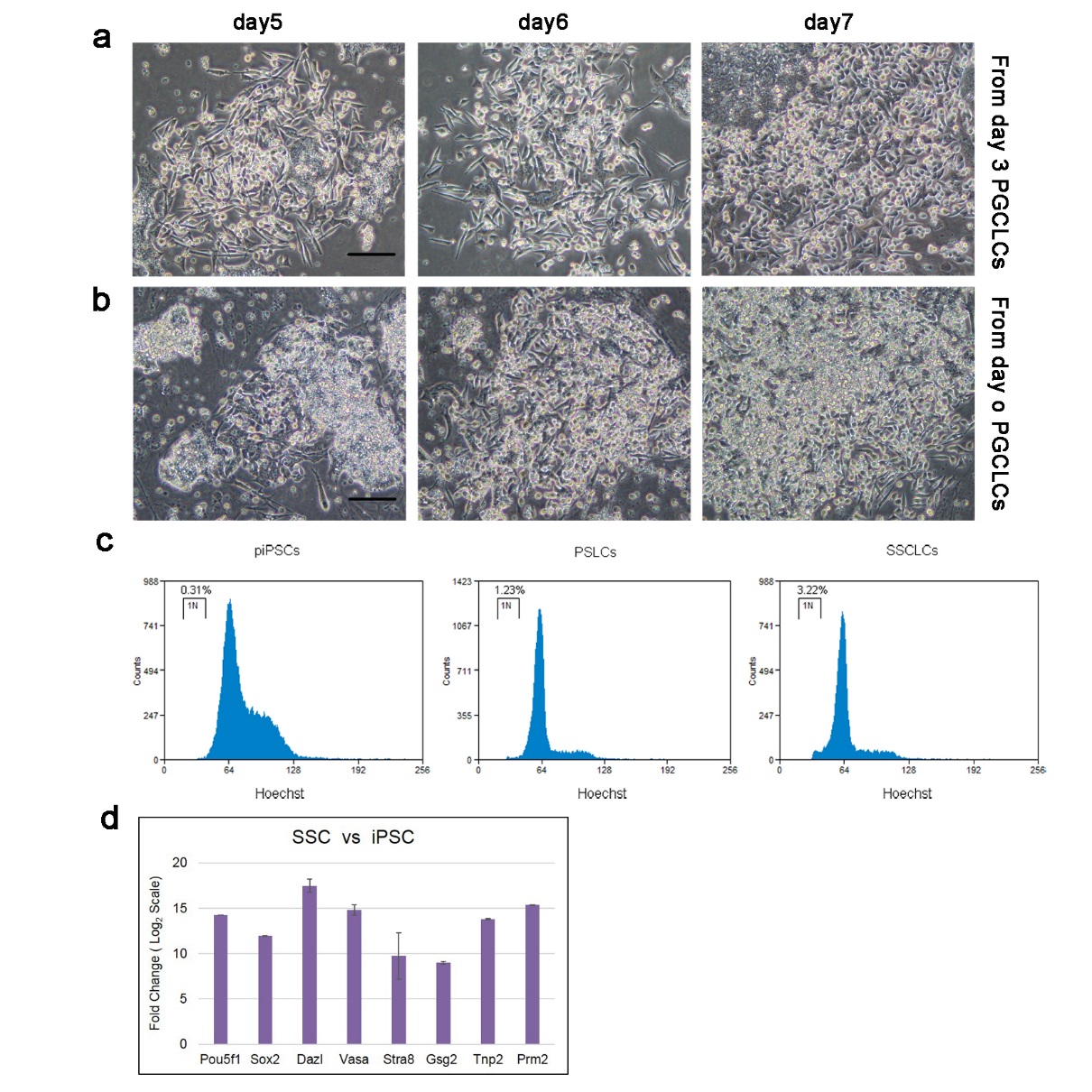


**Supplementary Figure 3. Biological characters of SSCLCs and SSCs**

(a and b) Cell morphology on day 5, 6 and 7 from day 3 PGCLCs (a) and day 0 PGCLCs (b) after SSCLCs induction. Scale bar, 100 μm.

(c) Flow cytometry analysis of DNA content revealing the presence of 0.31%, 1.23% and 3.22% haploid cells in piPSCs, PSLCs and SSCLCs, respectively.

(d) Gene expression dynamics in SSCs from pig testes by quantitative RT-PCR. The piPSCs were used as the control. Error bars indicate SDs from technical replicates.


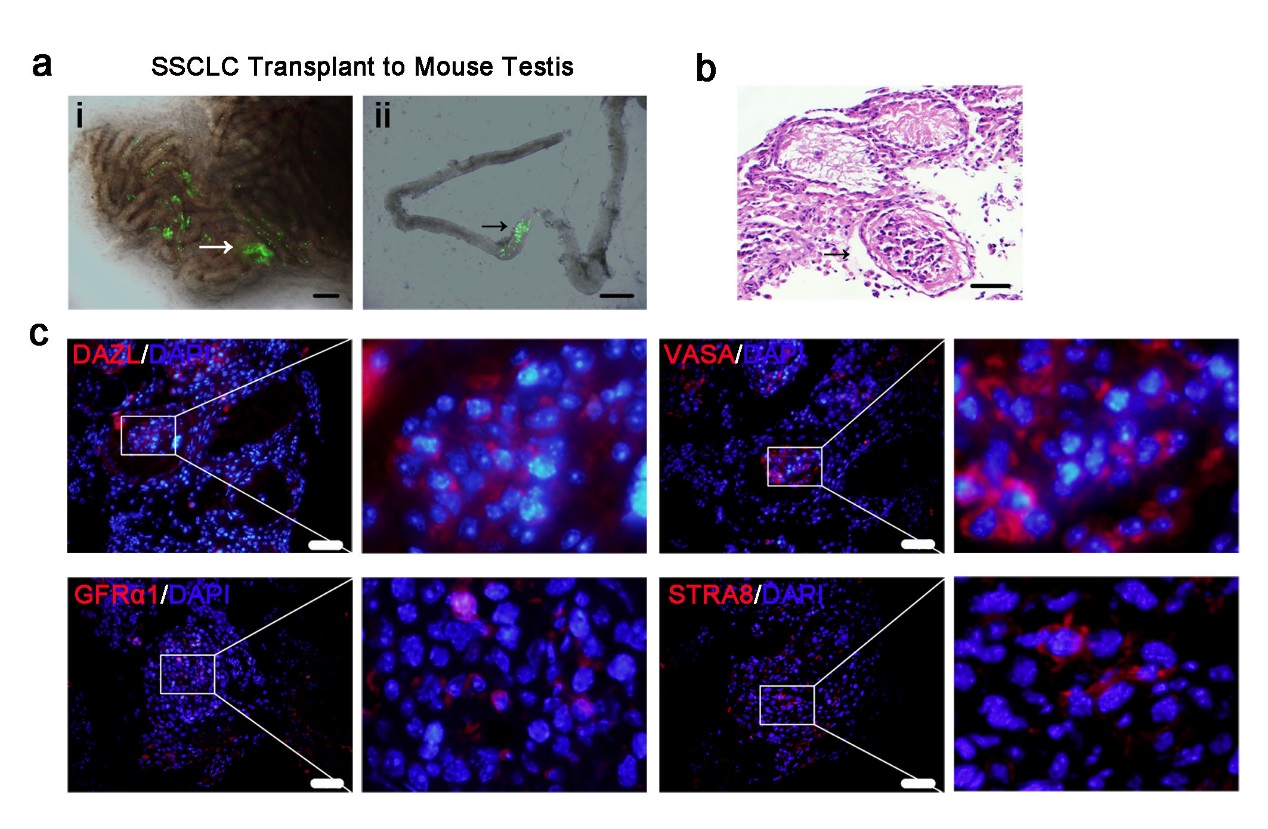


**Supplementary Figure 4. Transplantation of SSCLCs into busulfan-treated mouse testes**

(a) The seminiferous tubules transplanted with induced SSCLCs. The white arrow indicates donor-derived clusters of tubules. The black arrow indicates the individual tubule. Scale bar, 500 μm (i and ii).

(b) HE staining of testes after transplantation of SSCLCs. Scale bar, 50 μm.

(c) Immunohistochemical analysis of testis xenografts from SSCLCs. Cross sections were immunostained for DAZL, VASA, GFRα1 and STRA8. Nuclei were stained with DAPI (Blue). Scale bar, 50 μm.


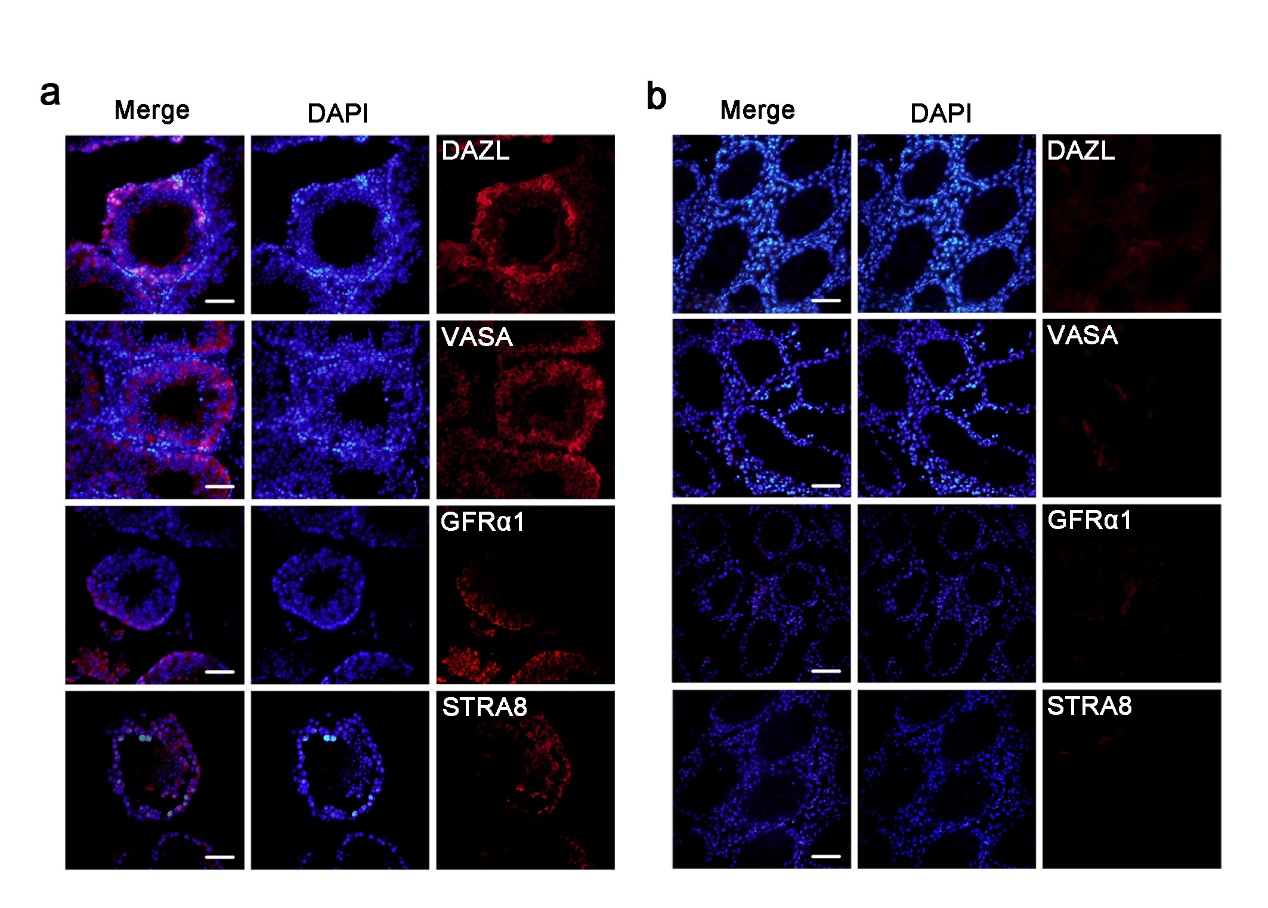


**Supplementary Figure 5. Immunofluorescence staining of positive and negative control of mouse testes**

(a and b) Immunofluorescence staining of positive (a) and negative (b) control of mouse testes for DAZL, VASA, GFRα1 and STRA8. Nuclei were stained with DAPI (Blue). Scale bar, 50 μm.


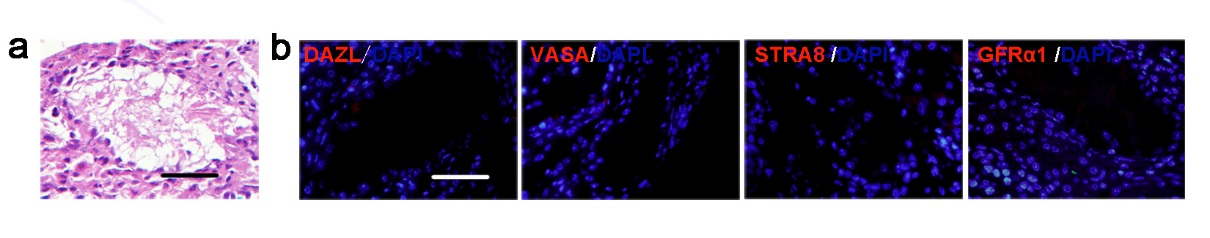


**Supplementary Figure 6. HE and Immunofluorescence staining of seminiferous tubules without ZsGreen cells**

(a) HE staining of seminiferous tubules without ZsGreen cells.Scale bar, 50 μm.

(b) Immunofluorescence staining of DAZL, VASA, STRA8 and GFRα1 in the seminiferous tubules without ZsGreen cells. Nuclei were stained with DAPI (Blue). Scale bar, 50 μm.

**Supplementary Methods**

**Isolation of SSCs from pig testes**

Pig testes were surgically removed and placed in DPBS (GIBCO), supplemented with penicillin/streptomycine (Gibico). The tissues were minced and then digested with Collagenase IV (Gibco) in 37 ℃ water bath for 20 min. After that, the seminiferous tubules were digested with Trypsin-EDTA (Gibico) and DNase (Sigma) in 37 ℃ water bath for 3 min. Lyse red blood cells in the lysis buffer (Beyotime) in room temperature for 3 min. SSCs were isolated by the method of differential adhesion (15min-20min-25min-30min-35min-40min）[^1^](#_ENREF_1). Collect the cell suspension without gelatin adhension.

**Flow Cytometry Analysis**

SSCs from pig testes were suspended in DPBS with 1% BSA, filtered through a 40-μm falcon (BD), and incubated with THY1 (a conjugated primary antibody, BD) antibody for 30 min. For ploidy analysis, cells were digested with Tryple and the single cell suspensions were stained with 10 mg/ml Hoechst 33342 for 50 min, washed three times with DPBS and filtered through a 40-μm falcon. FACS analysis[^3^](#_ENREF_3) was performed using the Moflo-XDP (Beckman).

**References:**

1 Zheng, Y. *et al.* THY1 is a surface marker of porcine gonocytes. *Reproduction, fertility, and development* **26**, 533-539, doi:10.1071/RD13075 (2014).

2 Maki, C. B. *et al.* Phenotypic and molecular characterization of spermatogonial stem cells in adult primate testes. *Hum Reprod* **24**, 1480-1491, doi:10.1093/humrep/dep033 (2009).

3 Pei, Y. *et al.* Improvement in Mouse iPSC Induction by Rab32 Reveals the Importance of Lipid Metabolism during Reprogramming. *Scientific reports* **5**, 16539, doi:10.1038/srep16539 (2015).
